# Microbial Interaction between Lactiplantibacillus plantarum and Saccharomyces cerevisiae: Transcriptome Level Mechanism of Cell-Cell Antagonism

**DOI:** 10.1128/spectrum.01433-22

**Published:** 2022-08-18

**Authors:** Junyan Liu, Teng-Yi Huang, Gongliang Liu, Yanrui Ye, Thanapop Soteyome, Gamini Seneviratne, Gengsheng Xiao, Zhenbo Xu, Birthe V. Kjellerup

**Affiliations:** a College of Light Industry and Food Science, Guangdong Provincial Key Laboratory of Lingnan Specialty Food Science and Technology, Academy of Contemporary Agricultural Engineering Innovations, Zhongkai University of Agriculture and Engineering, Guangzhou, China; b Key Laboratory of Green Processing and Intelligent Manufacturing of Lingnan Specialty Food, Ministry of Agriculture, Guangzhou, China; c Department of Laboratory Medicine, the Second Affiliated Hospital of Shantou University Medical College, Shantou, Guangdong, China; d School of Biology and Biological Engineering, South China University of Technology, Guangzhou, China; e Home Economics Technology, Rajamangala University of Technology Phra Nakhon, Bangkok, Thailand; f National Institute of Fundamental Studies, Kandy, Sri Lanka; g School of Food Science and Engineering, Guangdong Province Key Laboratory for Green Processing of Natural Products and Product Safety, Engineering Research Center of Starch and Vegetable Protein Processing Ministry of Education, South China University of Technology, Guangzhou, China; h Research Institute for Food Nutrition and Human Health, Guangzhou, China; i Department of Civil and Environmental Engineering, University of Maryland, College Park, Maryland, USA; University of Guelph

**Keywords:** microbial interaction, *L. plantarum*, *S. cerevisiae*, transcriptome, food

## Abstract

Lactiplantibacillus plantarum and Saccharomyces cerevisiae are frequently co-isolated in food, although playing different roles. This study aimed at investigating the microbial interaction between L. plantarum and S. cerevisiae, especially cell-cell direct interaction and their mechanism. Cell-cell and supernatant-cell coculture models were set up, with CFU counting, OD_600_ measurement, optical and atomic force microscopy performed to examine the growth and morphology of L. plantarum and S. cerevisiae cells. In cell-cell coculture model, L. plantarum cells inhibited S. cerevisiae growth (inhibition rate ~80%) with its own growth pattern unaffected. Cell-cell aggregation happened during coculture with surface roughness changed and partial S. cerevisiae cell lysis. Mature (24 h) L. plantarum cell-free culture supernatant showed inhibition (35%-75%) on S. cerevisiae growth independent of pH level, while supernatant from L. plantarum-S. cerevisiae coculture showed relatively stronger inhibition. Upon transcriptomics analysis, hypothesis on the mechanism of microbial interaction between L. plantarum and S. cerevisiae was demonstrated. When L. plantarum cell density reached threshold at 24 h, all genes in *lamBDCA* quorum sensing (QS) system was upregulated to potentially increase adhesion capability, leading to the aggregation to S. cerevisiae cell. The downregulation of whole basic physiological activity from DNA to RNA to protein, cell cycle, meiosis, and mitogen-activated protein kinase (MAPK) signaling pathways, as well as growth maintenance essential genes *ari1*, *skg6*, and *kex2*/*gas1* might induce the decreased growth and proliferation rate and partial death of S. cerevisiae cells in coculture.

**IMPORTANCE** L. plantarum and S. cerevisiae are frequently co-isolated in food, although playing different roles. The co-existence of L. plantarum and S. cerevisiae could result in variable effects, raising economic benefits and safety concerns in food industry. Previous research has reported the microbial interaction between L. plantarum and S. cerevisiae mainly rely on the signaling through extracellular metabolites. However, cell-cell aggregation has been observed with mechanism remain unknown. In the current study, the microbial interaction between L. plantarum and S. cerevisiae was investigated with emphasis on cell-cell direct interaction and further in-depth transcriptome level study showed the key role of *lamBDCA* quorum sensing system in L. plantarum. The results yield from this study demonstrated the antagonistic effect between L. plantarum and S. cerevisiae.

## INTRODUCTION

Lactiplantibacillus plantarum and cerevisiae are frequently co-isolated in traditional fermented food, including kefir (1, 2), wine (3, 4), beer (5, 6), and sourdoughs (7, 8). Mixed cultures of L. plantarum and S. cerevisiae had been employed to develop novel fermented beverages, improving the flavor and quality of the traditional fermented beverages (5, 9, 10). However, undesirable appearance of exotic microorganism in single species fermented food would cause unpredictable consequences (6, 11, 12). Beer spoilage had been reported to be caused by L. plantarum-induced premature yeast flocculation with physical interactions, while the spoilage of yogurt had been attributed to the production of carbon dioxide by yeasts (13). Thus, understanding on the potential interaction between L. plantarum and S. cerevisiae is of importance to guide the proper application of dual-species starter culture and potential control of harmful factors.

The interaction between L. plantarum and S. cerevisiae had been reported to mainly rely on the signaling through extracellular metabolites including lactic acid, fatty acid, ethanol, sulfur dioxide, and extracellular polysaccharides (14–17). S. cerevisiae could stimulate L. plantarum growth by secreting nutritional factors and consuming oxygen to form partial anaerobic environment (16). In L. plantarum-S. cerevisiae co-fermentation system, L. plantarum continuously produced lactic acid to reduce environmental pH, thus cells in stationary phase maintained intracellular pH balance by reducing intracellular proton concentration through amino acid metabolism (16). While S. cerevisiae adjusted its metabolism by secretion amino acids through efflux pumps in nitrogen source abundance environment, to enhance the growth and metabolism of L. plantarum in acidic conditions (10, 18). The excretion of sulfur dioxide by S. cerevisiae might be essential for the higher mortality in L. plantarum during winemaking (17).

The co-aggregation of L. plantarum with S. cerevisiae had also been observed with the mechanism remaining unclear (19), which was previously predicted to relate to mannose-specific adhesin and surface layer proteins (19, 20). Their co-aggregation is closely associated with the quality and safety of fermented food, including beer and kefir. In the beer industry, S. cerevisiae individual flocculation contributed to be separated from fermentation production after the available sugar was exhausted (21). However, premature yeast flocculation was induced by L. plantarum for their co-aggregation capacity, which reduced surface contact with the substrate to decrease ethanol production (14, 22).

Thus, the co-existence of L. plantarum and S. cerevisiae could result in variable effects depending on their roles in various food systems. As the interaction between L. plantarum and S. cerevisiae has raised additional economic benefits and safety concerns in food industry, the comprehensive interaction mechanism should be revealed. In this study, the microbial interaction between representative L. plantarum and S. cerevisiae was investigated with emphasis on cell-cell direct interaction and further transcriptome level mechanism was elucidated.

## RESULTS

### L. plantarum inhibits S. cerevisiae growth with its own growth pattern unaffected.

**FIG 1:**
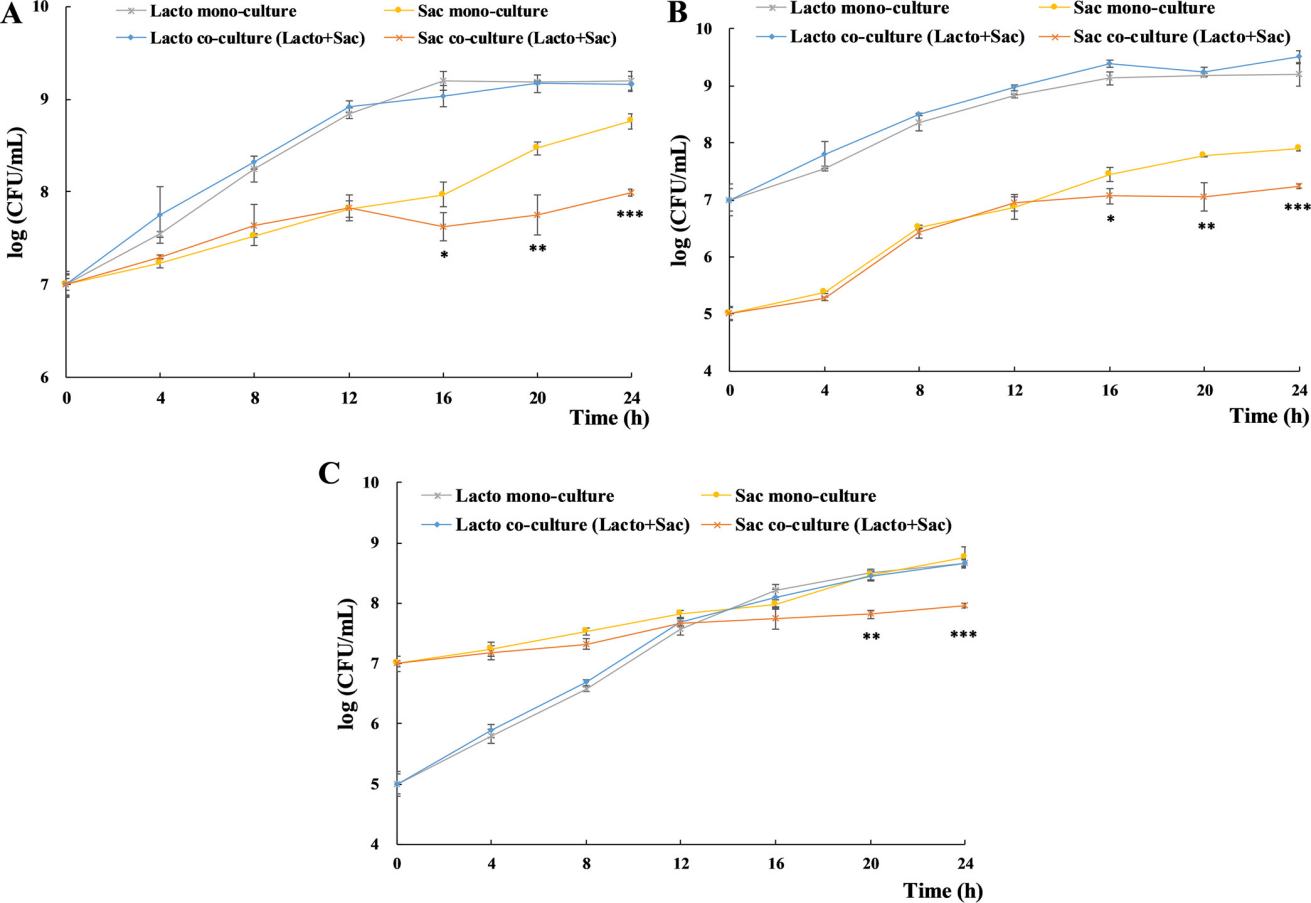
The growth curves of L. plantarum and S. cerevisiae in coculture and monococulture under different initial concentrations. All experiments were conducted in biological triplicates. *, *P* < 0.05; **, *P* < 0.01; ***, *P* < 0.001. (A) Initial concentration at 10^7^ CFU/mL for L. plantarum and 10^7^ CFU/mL for S. cerevisiae (10^7^ Lacto + 10^7^ Sac). (B) Initial concentration at 10^7^ CFU/mL for L. plantarum and 10^5^ CFU/mL for S. cerevisiae (10^7^ Lacto + 10^5^ Sac). (C) Initial concentration at 10^5^ CFU/mL for L. plantarum and 10^7^ CFU/mL for S. cerevisiae (10^5^ Lacto + 10^7^ Sac).

In order to investigate the microbial interaction between S. cerevisiae and L. plantarum cells, we firstly examined their growth curve in cell-cell coculture model, with the growth in monococulture as control. Considering the complication of food processing and storage environments where S. cerevisiae and L. plantarum co-exist, three groups (10^7^ Lacto + 10^5^ Sac, 10^7^ Lacto + 10^7^ Sac, 10^5^ Lacto + 10^7^ Sac) with initial concentration ratio at 100:1, 1:1, and 1:100 were included to mimic the difference in their relative cell numbers. Additionally, blank control with distilled H_2_O to replace MRS broth in S. cerevisiae monococulture control group and to replace yeast extract-peptone-dextrose (YPD) broth in L. plantarum monococulture control group were included, respectively, to examine the influence of un-inoculated medium and nutrient difference in experimental groups. Insignificant difference was observed in both blank controls (data not shown). With the same initial concentration (10^7^ CFU/mL, 1:1), S. cerevisiae and L. plantarum cell numbers were uninfluenced by each other within 12 h. However, significant lower cell number of S. cerevisiae in coculture was determined since 16 h compared with that in monococulture (Fig. 1A), indicating the inhibition of L. plantarum cells on the growth of S. cerevisiae. Inhibition rate of 55.31% (5.19 × 10^7^ CFU/mL), 81.07% (2.40 × 10^8^ CFU/mL), and 83.00% (4.81 × 10^8^ CFU/mL) were examined at 16 h, 20 h, and 24 h, respectively. When the initial concentrations (100:1) of S. cerevisiae and L. plantarum were 10^7^ CFU/mL and 10^5^ CFU/mL, respectively, similar growth patterns were determined (Fig. 1B). In the group with initial concentration ratio at 1:100, the cell number of S. cerevisiae in coculture was not significantly lower than that in monococulture until 20 h (Fig. 1C). While in the three groups, the growth of L. plantarum remained stable either with or without the presence of S. cerevisiae cells. In summary, with the same amount of L. plantarum cells, the difference in S. cerevisiae initial cell number had no effect on the inhibition pattern, while with the same amount of S. cerevisiae cells, fewer L. plantarum cells delayed the inhibition on S. cerevisiae growth. Thus, L. plantarum potentially focused on its own growth within logarithmic phase to reach a certain cell number and somehow started to inhibit the growth of S. cerevisiae thereafter. Additionally, similar inhibition effect was observed in a few other *Lactobacillus* spp. (L. casei, L. acetotolerans, L. linderi, and L. brevis) and *Pediococcus* spp. (P. damnonsus and P. acidilactici) strains.

### Decreased surface roughness of S. cerevisiae cells with the surrounding of L. plantarum cells.

**FIG 2:**
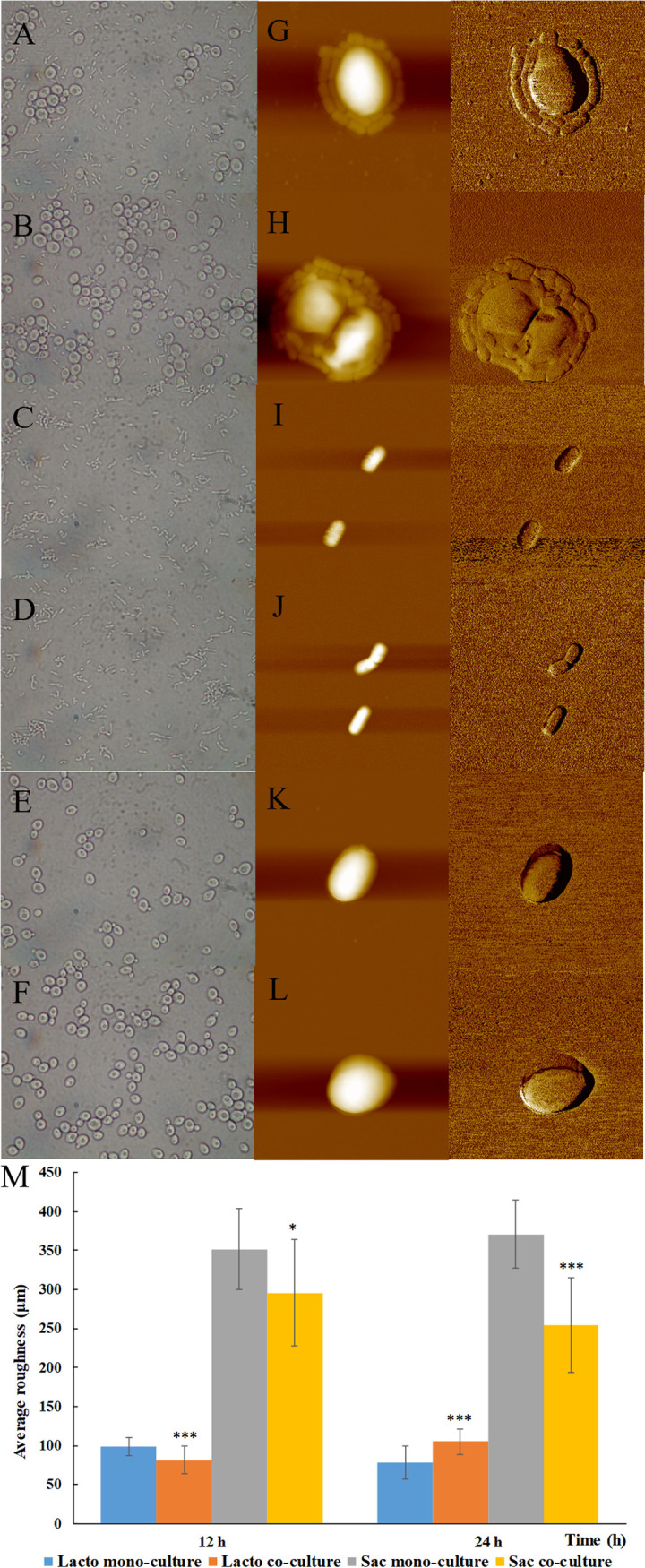
The morphology from optical microscopy (A-F) and atomic force microscopy (G-L) and surface roughness (M) of L. plantarum and S. cerevisiae cells in mono- and co-culturecoculture. All experiments were conducted in biological triplicate. *, *P* < 0.05; **, *P* < 0.01; ***, *P* < 0.001. A, G: L. plantarum and S. cerevisiae co-culturecoculture for 12 h (LhSc_12h); B, H: L. plantarum and S. cerevisiae co-culturecoculture for 24 h (LhSc_24h); C, I: L. plantarum mono-culturemonococulture for 12 h (Lh_12h); D, J: L. plantarum mono-culturemonococulture for 24 h (Lh_24h); E, K: S. cerevisiae mono-culturemonococulture for 12 h (Sc_12h); F, L: S. cerevisiae mono-culturemonococulture for 24 h (Sc_24h).

The morphology of L. plantarum and S. cerevisiae cells during mono and coculture at 12 h and 24 h was monitored by optical microscopy and atomic force microscope (AFM) (Fig. 2). Cell-cell direct contact was observed in coculture, with L. plantarum cells surrounding S. cerevisiae cells (Fig. 2A and B). According to AFM images, L. plantarum and S. cerevisiae cells were intact in monococulture at 12 h and 24 h (Fig. 2C to F) and coculture at 12 h (Fig. 2A). However, cell lysis was observed for S. cerevisiae cells in coculture at 24 h (Fig. 2B). Cell surface roughness was further calculated based on 3D images from AFM (Fig. 2G). In mono- and coculture, the surface roughness of S. cerevisiae cells was more than 3-fold higher than that of L. plantarum cells. At 12 h, the surface roughness of L. plantarum and S. cerevisiae cells in coculture were significantly lower than those in monococulture. According to previous studies, the increase on surface roughness is related to the shrunk of cell wall or accumulation of cell surface metabolites. However, in this study, cell wall shrinking was not observed. As each growth was not influenced, L. plantarum and S. cerevisiae might lower partial metabolism to maintain growth rate, resulting in less metabolite accumulation. At 24 h, the surface roughness of L. plantarum cells significantly increased in coculture, indicating more metabolites accumulation from 12 h to 24 h. However, the surface roughness of S. cerevisiae cells reduced in coculture at 24 h, possibly influenced by the surrounding of L. plantarum cells.

### Metabolites from L. plantarum mature culture inhibit S. cerevisiae growth.

**FIG 3:**
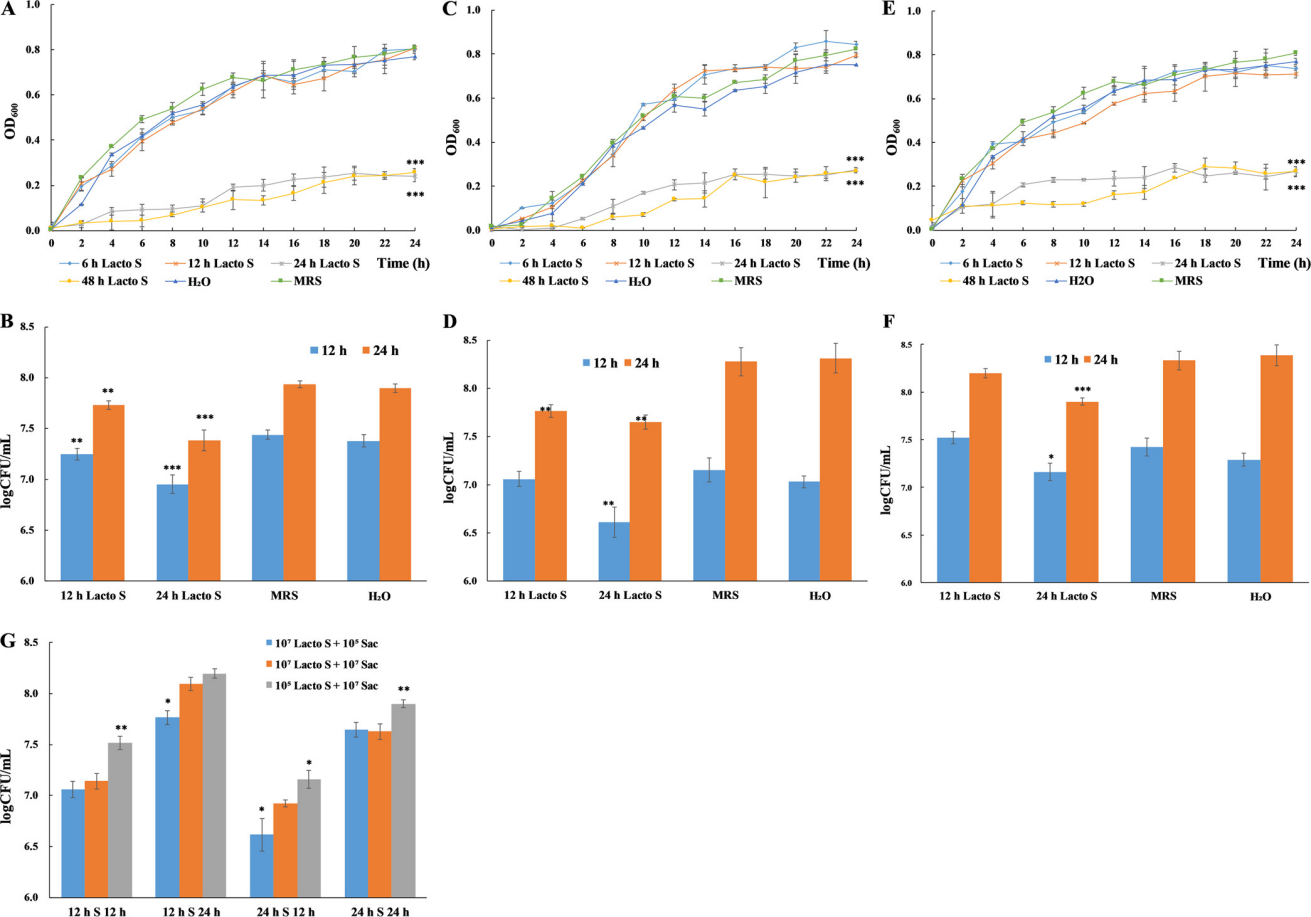
The effect of L. plantarum supernatant on S. cerevisiae growth under different initial concentrations determined by OD_600_ measurement (A, C, E) and CFU counting (B, D, F). The cell numbers of S. cerevisiae treated with 12 h L. plantarum supernatant (12 h S) and 24 h L. plantarum supernatant (24 h S) at 12 h and 24 h were compared in each group (G). All experiments were conducted in biological triplicate. *, *P* < 0.05; **, *P* < 0.01; ***, *P* < 0.001. A & B: Initial concentration at 10^7^ CFU/mL for L. plantarum with supernatant collected at 6 h (6 h Lacto S), 12 h (12 h Lacto S), 24 h (24 h Lacto S), and 48 h (48 h Lacto S), and 10^7^ CFU/mL for S. cerevisiae (10^7^ Lacto S + 10^7^ Sac). C & D: Initial concentration at 10^7^ CFU/mL for L. plantarum with supernatant collected and 10^5^ CFU/mL for S. cerevisiae (10^7^ Lacto S + 10^5^ Sac). E & F: Initial concentration at 10^5^ CFU/mL for L. plantarum with supernatant collected and 10^7^ CFU/mL for S. cerevisiae (10^5^ Lacto S + 10^7^ Sac).

To examine whether the inhibition effect of L. plantarum on S. cerevisiae growth is due to the metabolites produced by L. plantarum, cell-free culture supernatant (Lacto S) was collected upon culturing L. plantarum for 6 h, 12 h, 24 h, and 48 h, respectively, and cocultured with S. cerevisiae cells. In addition, three groups (10^7^ Lacto S + 10^7^ Sac, 10^7^ Lacto S + 10^5^ Sac, 10^5^ Lacto S + 10^7^ Sac) with different initial concentrations were included. Additionally, blank control with distilled H_2_O to replace MRS broth in mono-supernatant control group was included to examine the influence of nutrient difference in experimental groups. Insignificant difference in the growth of S. cerevisiae between blank control (represent the minimum additional nutrient) and mono-supernatant control (represent the maximum additional nutrient) indicates nutrient in S. cerevisiae culture was abundant thus nutrient depletion was not a factor contributing to the change in growth (Fig. 3). Among all experimental groups, 24 h and 48 h L. plantarum supernatant showed significant inhibition on S. cerevisiae growth, regardless of initial concentrations (Fig. 3A, C and E). The cell numbers at 12 h and 24 h were assessed by CFU counting and inhibition rates were measured accordingly (Fig. 3B, D and F). In group 10^7^ Lacto S + 10^7^ Sac, the inhibition rate of 12 h L. plantarum supernatant on S. cerevisiae growth was 35.75% (9.78 × 10^6^ CFU/mL) and 37.41% (3.20 × 10^7^ CFU/mL) at 12 h and 24 h, respectively, although OD_600_ remained unchanged (Fig. 3B). It implied partial S. cerevisiae cell death during coculture with 12 h L. plantarum supernatant. While 24 h L. plantarum supernatant showed 67.23% (1.84 × 10^7^ CFU/mL) and 71.89% (6.15 × 10^7^ CFU/mL) inhibition on S. cerevisiae growth at 12 h and 24 h, respectively. Similar inhibition pattern was identified in group 10^7^ Lacto S + 10^5^ Sac (Fig. 3D). However, in group 10^5^ Lacto S + 10^7^ Sac, only 24 h L. plantarum supernatant showed inhibition (45.30% at 12 h and 62.88% at 24 h) on S. cerevisiae growth.

Collectively, initial concentration of L. plantarum might influence the metabolites accumulation efficiency in early time points (12 h). With an initial concentration at 10^7^ CFU/mL, the supernatant collected from 12 h L. plantarum culture could induce partial death of S. cerevisiae cells but not its growth. However, with an initial concentration at 10^5^ CFU/mL, 12 h L. plantarum culture supernatant could neither induce cell death nor growth of S. cerevisiae. Upon reaching stationary phase (24 h), L. plantarum has accumulated abundant metabolites to inhibit the growth of S. cerevisiae regardless of initial concentration, which somewhat related to inhibition rate. In addition, S. cerevisiae survival rate was concentration dependent (Fig. 3G).

### Metabolites from L. plantarum -S. cerevisiae coculture show stronger inhibition on S. cerevisiae growth.

**FIG 4:**
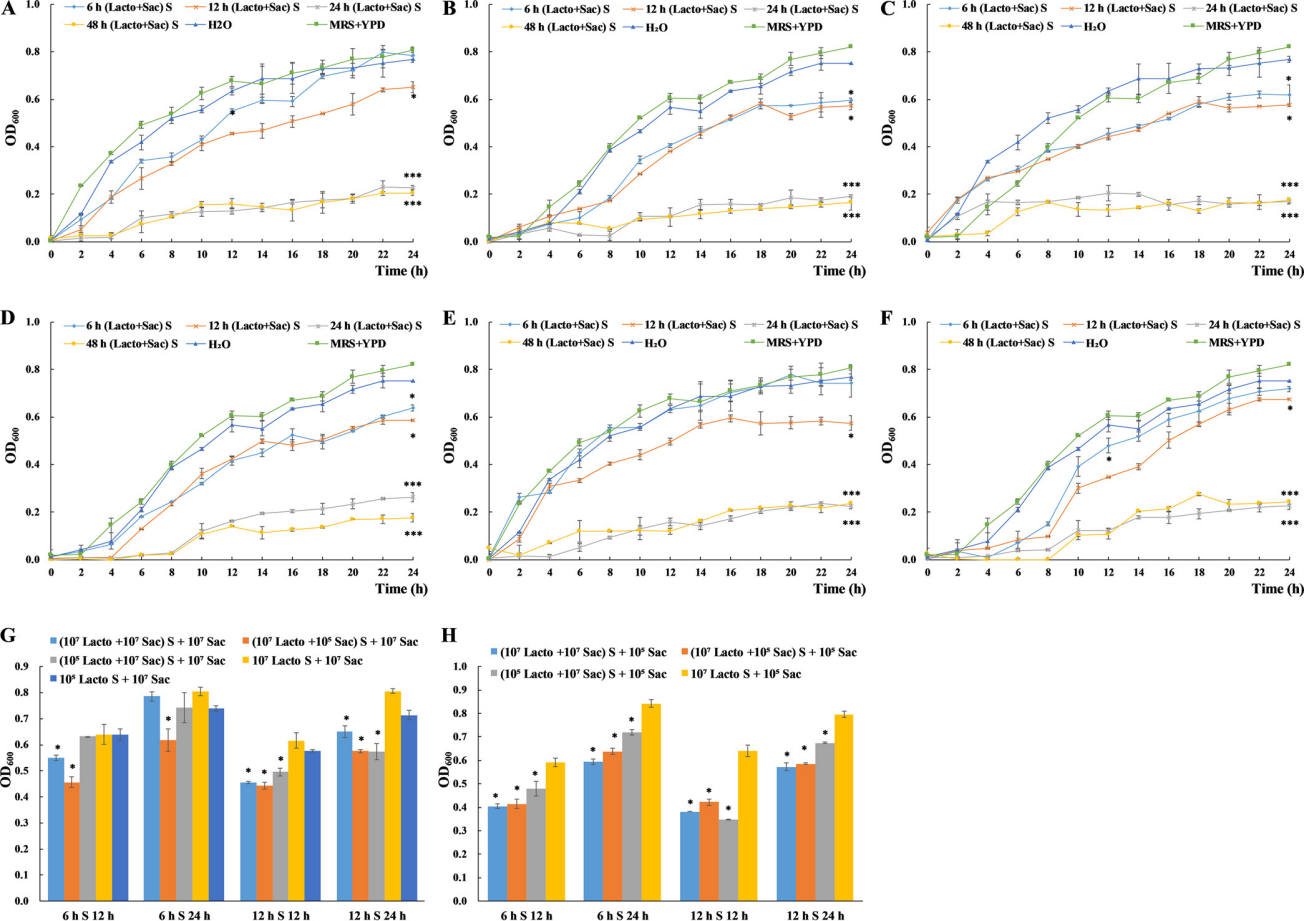
The effect of coculture supernatant on S. cerevisiae growth under different initial concentrations determined by OD_600_ measurement (A) to (F). The OD_600_ values of S. cerevisiae cultures with initial concentrations at 10^7^ CFU/mL (G) and 10^5^ CFU/mL (H) treated with 6 h L. plantarum -S. cerevisiae coculture supernatant (6 h S) and 12 h coculture supernatant (12 h S) at 12 h and 24 h were compared in each group with L. plantarum monococulture supernatant treatment as control (G). All experiments were conducted in biological triplicates. *, *P* < 0.05; **, *P* < 0.01; ***, *P* < 0.001. (A) S. cerevisiae with an initial concentration at 10^7^ CFU/mL was treated with supernatants collected from the coculture of L. plantarum (10^7^ CFU/mL) and S. cerevisiae (10^7^ CFU/mL) ([10^7^ Lacto + 10^7^ Sac] S + 10^7^ Sac). (B) S. cerevisiae with an initial concentration at 10^5^ CFU/mL was treated with supernatants collected from the coculture of L. plantarum (10^7^ CFU/mL) and S. cerevisiae (10^7^ CFU/mL) ([10^7^ Lacto + 10^7^ Sac] S + 10^5^ Sac). (C) S. cerevisiae with an initial concentration at 10^7^ CFU/mL was treated with supernatants collected from the coculture of L. plantarum (10^7^ CFU/mL) and S. cerevisiae (10^5^ CFU/mL) ([10^7^ Lacto + 10^5^ Sac] S + 10^7^ Sac). (D) S. cerevisiae with an initial concentration at 10^5^ CFU/mL was treated with supernatants collected from the coculture of L. plantarum (10^7^ CFU/mL) and S. cerevisiae (10^5^ CFU/mL) ([10^7^ Lacto + 10^5^ Sac] S + 10^5^ Sac). (E) S. cerevisiae with an initial concentration at 10^7^ CFU/mL was treated with supernatants collected from the coculture of L. plantarum (10^5^ CFU/mL) and S. cerevisiae (10^7^ CFU/mL) ([10^5^ Lacto + 10^7^ Sac] S + 10^7^ Sac). (F) S. cerevisiae with an initial concentration at 10^5^ CFU/mL was treated with supernatants collected from the coculture of L. plantarum (10^5^ CFU/mL) and S. cerevisiae (10^7^ CFU/mL) ([10^5^ Lacto + 10^7^ Sac] S + 10^5^ Sac).

**FIG 5:**
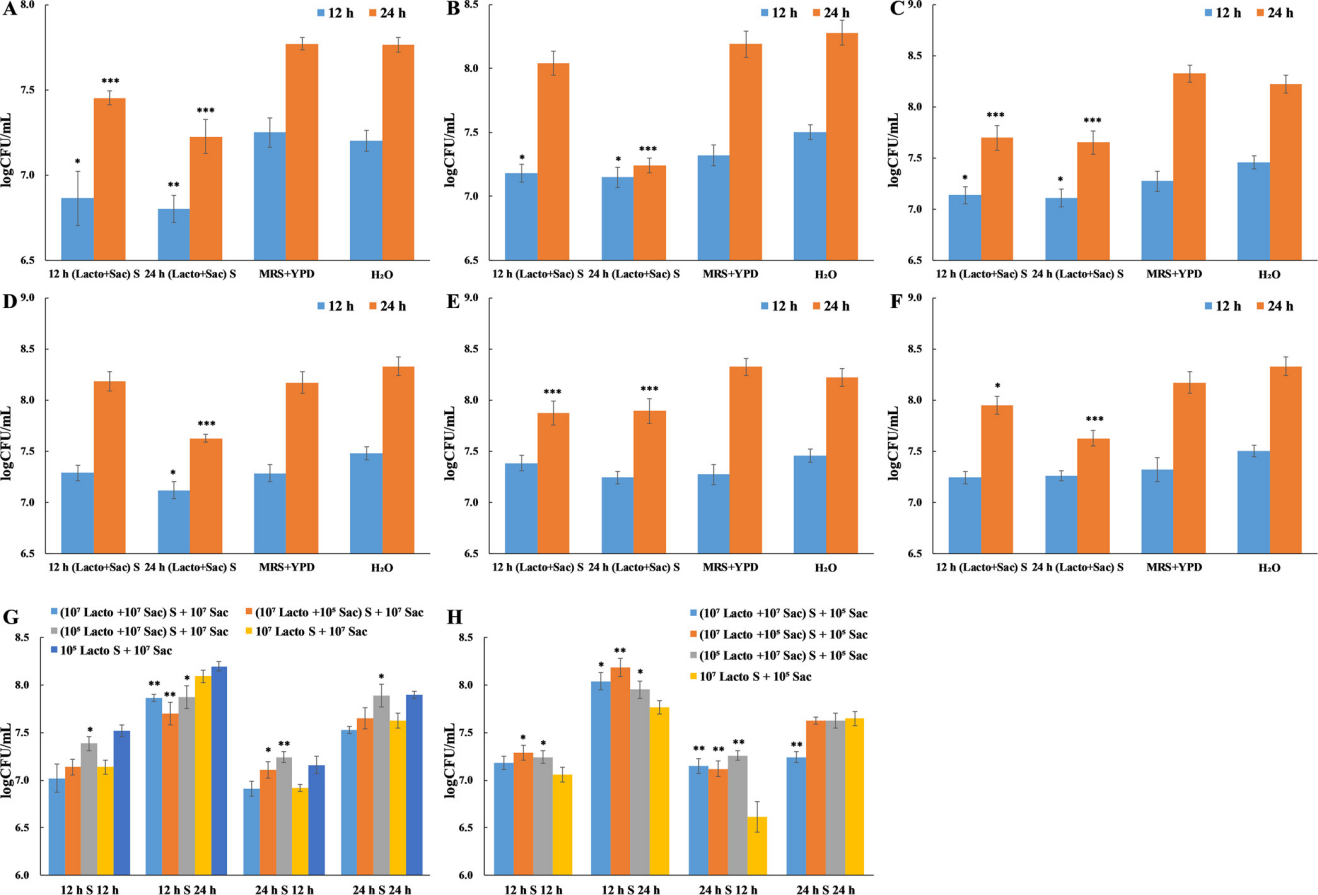
The effect of coculture supernatant on S. cerevisiae growth under different initial concentrations at 12 h and 24 h determined by CFU counting (A) to (F). The cell numbers of S. cerevisiae with initial concentrations at 10^7^ CFU/mL (G) and 10^5^ CFU/mL (H) treated with 12 h L. plantarum-S. cerevisiae coculture supernatant (12 h S) and 24 h coculture supernatant (24 h S) at 12 h and 24 h were compared in each group with L. plantarum monococulture supernatant treatment as control (G). All experiments were conducted in biological triplicates. *, *P* < 0.05; **, *P* < 0.01; ***, *P* < 0.001. (A) S. cerevisiae with an initial concentration at 10^7^ CFU/mL was treated with supernatants collected from the coculture of L. plantarum (10^7^ CFU/mL) and S. cerevisiae (10^7^ CFU/mL) ([10^7^ Lacto + 10^7^ Sac] S + 10^7^ Sac). (B) S. cerevisiae with an initial concentration at 10^5^ CFU/mL was treated with supernatants collected from the coculture of L. plantarum (10^7^ CFU/mL) and S. cerevisiae (10^7^ CFU/mL) ([10^7^ Lacto + 10^7^ Sac] S + 10^5^ Sac). (C) S. cerevisiae with an initial concentration at 10^7^ CFU/mL was treated with supernatants collected from the coculture of L. plantarum (10^7^ CFU/mL) and S. cerevisiae (10^5^ CFU/mL) ([10^7^ Lacto + 10^5^ Sac] S + 10^7^ Sac). (D) S. cerevisiae with an initial concentration at 10^5^ CFU/mL was treated with supernatants collected from the coculture of L. plantarum (10^7^ CFU/mL) and S. cerevisiae (10^5^ CFU/mL) ([10^7^ Lacto + 10^5^ Sac] S + 10^5^ Sac). (E) S. cerevisiae with an initial concentration at 10^7^ CFU/mL was treated with supernatants collected from the coculture of L. plantarum (10^5^ CFU/mL) and S. cerevisiae (10^7^ CFU/mL) ([10^5^ Lacto + 10^7^ Sac] S + 10^7^ Sac). (F) S. cerevisiae with an initial concentration at 10^5^ CFU/mL was treated with supernatants collected from the coculture of L. plantarum (10^5^ CFU/mL) and S. cerevisiae (10^7^ CFU/mL) ([10^5^ Lacto + 10^7^ Sac] S + 10^5^ Sac).

Considering the change in metabolism of L. plantarum and S. cerevisiae during coculture, cell-free culture supernatant from coculture ([Lacto+Sac] S) was collected upon coculturing for 6 h, 12 h, 24 h, and 48 h, respectively. S. cerevisiae cells were treated with coculture supernatant and growth curves were examined. Six groups ((10^7^ Lacto + 10^7^ Sac) S + 10^7^ Sac, (10^7^ Lacto + 10^7^ Sac) S + 10^5^ Sac, (10^7^ Lacto + 10^5^ Sac) S + 10^7^ Sac, (10^7^ Lacto + 10^5^ Sac) S + 10^5^ Sac, (10^5^ Lacto + 10^7^ Sac) S + 10^7^ Sac, (10^5^ Lacto + 10^7^ Sac) S + 10^5^ Sac) with different initial concentrations were included (Fig. 4 and 5). Additionally, blank control with distilled H_2_O to replace MRS-YPD broth in co-supernatant control group was included to examine the influence of nutrient difference in experimental groups. Insignificant difference in the growth of S. cerevisiae between blank control (represent the minimum additional nutrient) and co-supernatant control (represent the maximum additional nutrient) indicates nutrient in S. cerevisiae culture was abundant thus nutrient depletion was not a factor contributing to the change in growth (Fig. 3). In all experimental groups, the growth of S. cerevisiae was 70%–90% inhibited by 24 h and 48 h coculture supernatants, and to a less extent, 30%-50% inhibited by 12 h coculture supernatant (Fig. 4A to F and Fig. 5A to F), independent of initial concentration. In groups (10^7^ Lacto + 10^7^ Sac) S + 10^5^ Sac, (10^7^ Lacto + 10^5^ Sac) S + 10^7^ Sac, and (10^7^ Lacto + 10^5^ Sac) S + 10^5^ Sac, 6 h coculture supernatant could relatively inhibit the growth of S. cerevisiae in 24 h (Fig. 4B to D). In groups (10^7^ Lacto + 10^7^ Sac) S + 10^7^ Sac and (10^5^ Lacto + 10^7^ Sac) S + 10^5^ Sac, the growth of S. cerevisiae was partially (<12 h) inhibited by 6 h coculture supernatant (Fig. 4A and F). It indicated the metabolites from 6 h coculture supernatant differed depend on initial cell number.

The effect of 6 h and 12 h monococulture and coculture supernatants on the growth of S. cerevisiae were compared (Fig. 4G and H, Fig. 5G and H). With the same initial concentration, S. cerevisiae had significantly lower growth or survival rate in coculture supernatant treatment groups. Higher concentration in L. plantarum promoted the inhibition effect. Thus, the metabolism of L. plantarum and S. cerevisiae has changed during coculture potentially due to microbial interaction, leading to the accumulation of metabolites inhibiting the growth of S. cerevisiae.

### The inhibition of L. plantarum supernatant on S. cerevisiae growth is independent of pH value.

**FIG 6:**
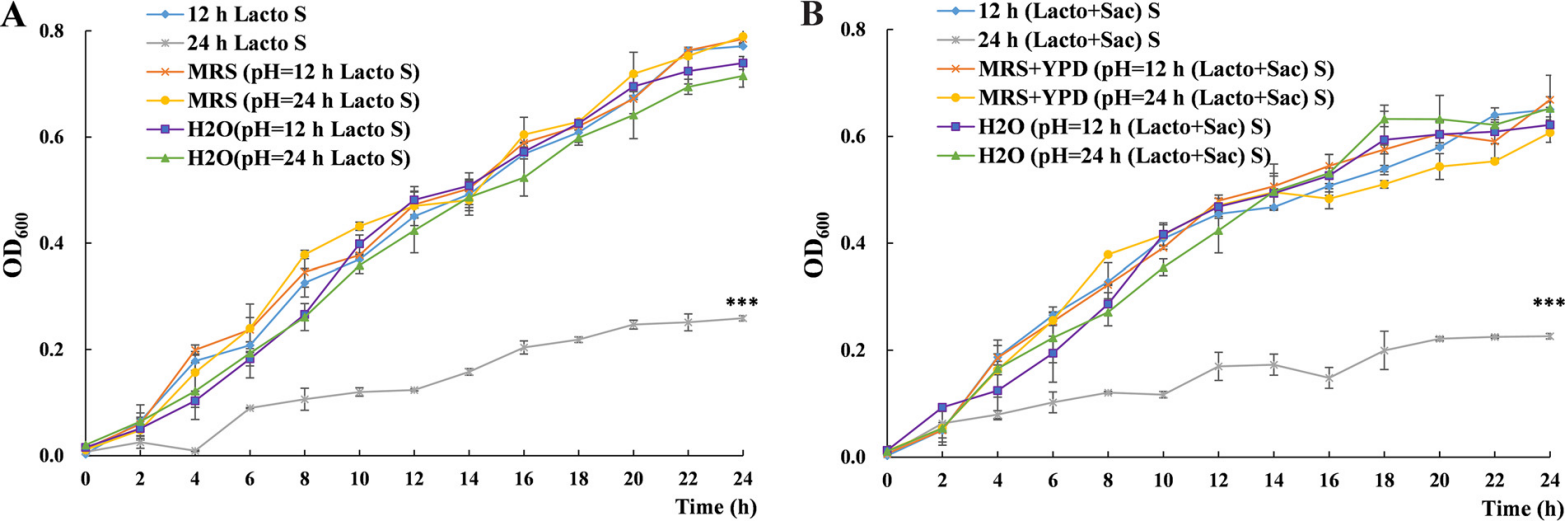
The effect of pH value comparing with monoculture (A) and coculture (B) supernatant on S. cerevisiae growth. All experiments were conducted in biological triplicates. *, *P* < 0.05; **, *P* < 0.01; ***, *P* < 0.001. MRS (pH = 12 h Lacto S): MRS medium with pH value adjusted to the same as 12 h L. plantarum monococulture supernatant, MRS (pH = 24 h Lacto S): MRS medium with pH value adjusted to the same as 24 h L. plantarum monococulture supernatant, MRS+YPD (pH = 12 h [Lacto+Sac] S): MRS and YPD media mixture with pH value adjusted to the same as 12 h L. plantarum -S. cerevisiae coculture supernatant, MRS+YPD (pH = 24 h [Lacto+Sac] S): MRS and YPD media mixture with pH value adjusted to the same as 24 h L. plantarum -S. cerevisiae coculture supernatant.

Acid production is one of the major characteristics of lactic acid bacteria, generating an unfavorable low pH environment for some other microorganisms. The pH value of L. plantarum monococulture supernatant reduced to 4.17 at 12 h and 3.88 at 24 h, which were significantly lower than culturing medium (pH = 5.64). Similarly, the pH value of L. plantarum*-*S. cerevisiae coculture supernatant reduced to 4.23 at 12 h and 3.62 at 24 h. To identify whether lowered pH value is the key factor contributing to the inhibition on S. cerevisiae growth, S. cerevisiae cells were cultured in media (represent the maximum additional nutrient) and H_2_O (represent the minimum additional nutrient) with pH values adjusted to the same as mono- and coculture supernatants, respectively. Surprisingly, solely reducing the pH value of either culturing media or H_2_O was unable to inhibit the growth of S. cerevisiae to the extent of 24 h mono- or coculture supernatants (Fig. 6). Also, no significant difference was observed in pH adjusted media and blank control groups. Thus, acid environment generated by L. plantarum was not the key factor inducing its inhibition effect on S. cerevisiae growth.

### General features of the transcriptome profile of L. plantarum.

Considering above phenotypes, cell-cell coculture model and 12 h (no inhibition) and 24 h (significant inhibition) were selected to further explore the transcriptomics level changes of both L. plantarum and S. cerevisiae during microbial interaction. Six samples including L. plantarum monococulture at 12 h (Lh_12h) and 24 h (Lh_24h), monococulture of S. cerevisiae at 12 h (Sc_12h) and 24 h (Sc_24h), and coculture at 12 h (LhSc_12h) and 24 h (LhSc_24h) were adapted to RNA-seq in biological triplicates. RNA isolation, library construction, sequencing, and bioinformatics analysis on samples LhSc_12h and LhSc_24h were conducted twice with different strategies to achieve both transcriptome profiles of L. plantarum and S. cerevisiae separately in coculture.

**FIG 7:**
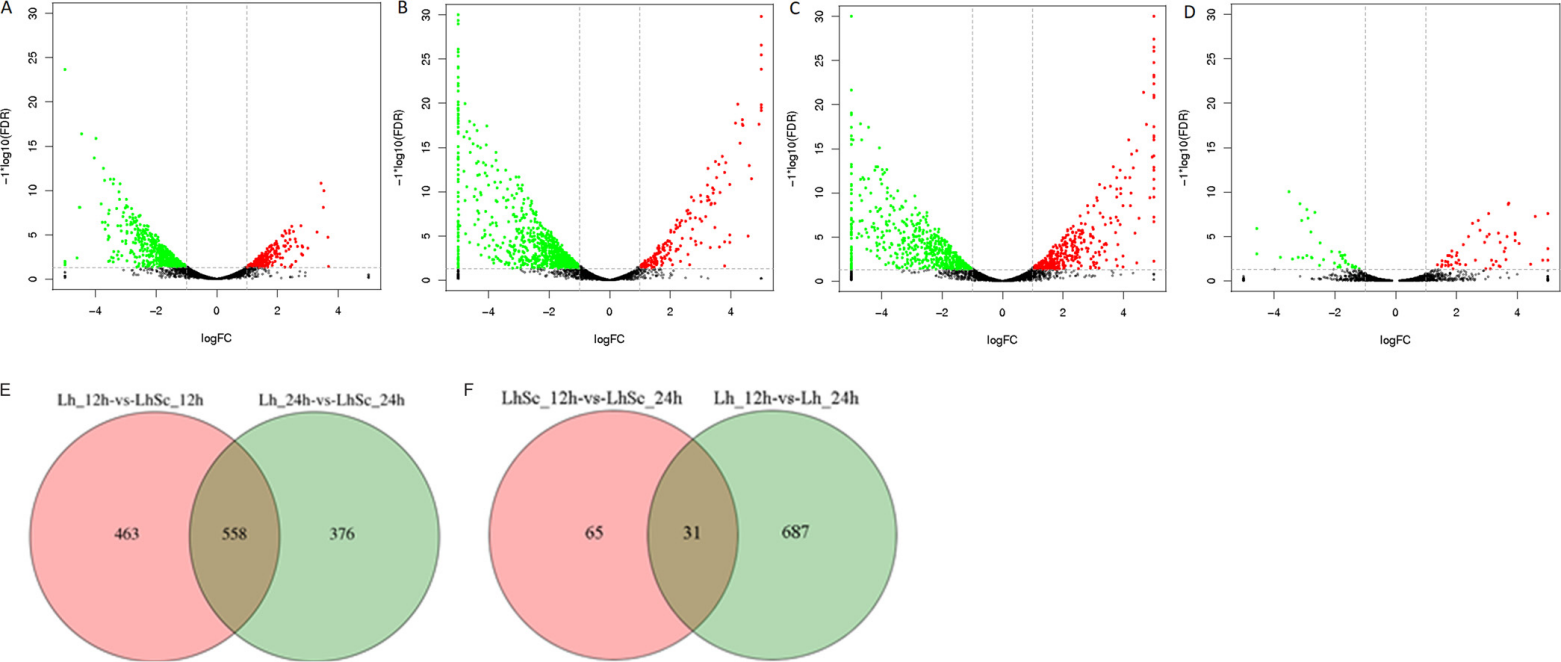
Volcano plots (A) to (D) and Venn map (E) to (F) of L. plantarum comparison groups. (A) Lh_12h vs Lh_24h, (B) Lh_12h vs LhSc_12h, (C) Lh_24h vs LhSc_24h, (D) LhSc_12h vs LhSc_24h. RNA-seq was conducted in biological triplicates.

**FIG 8:**
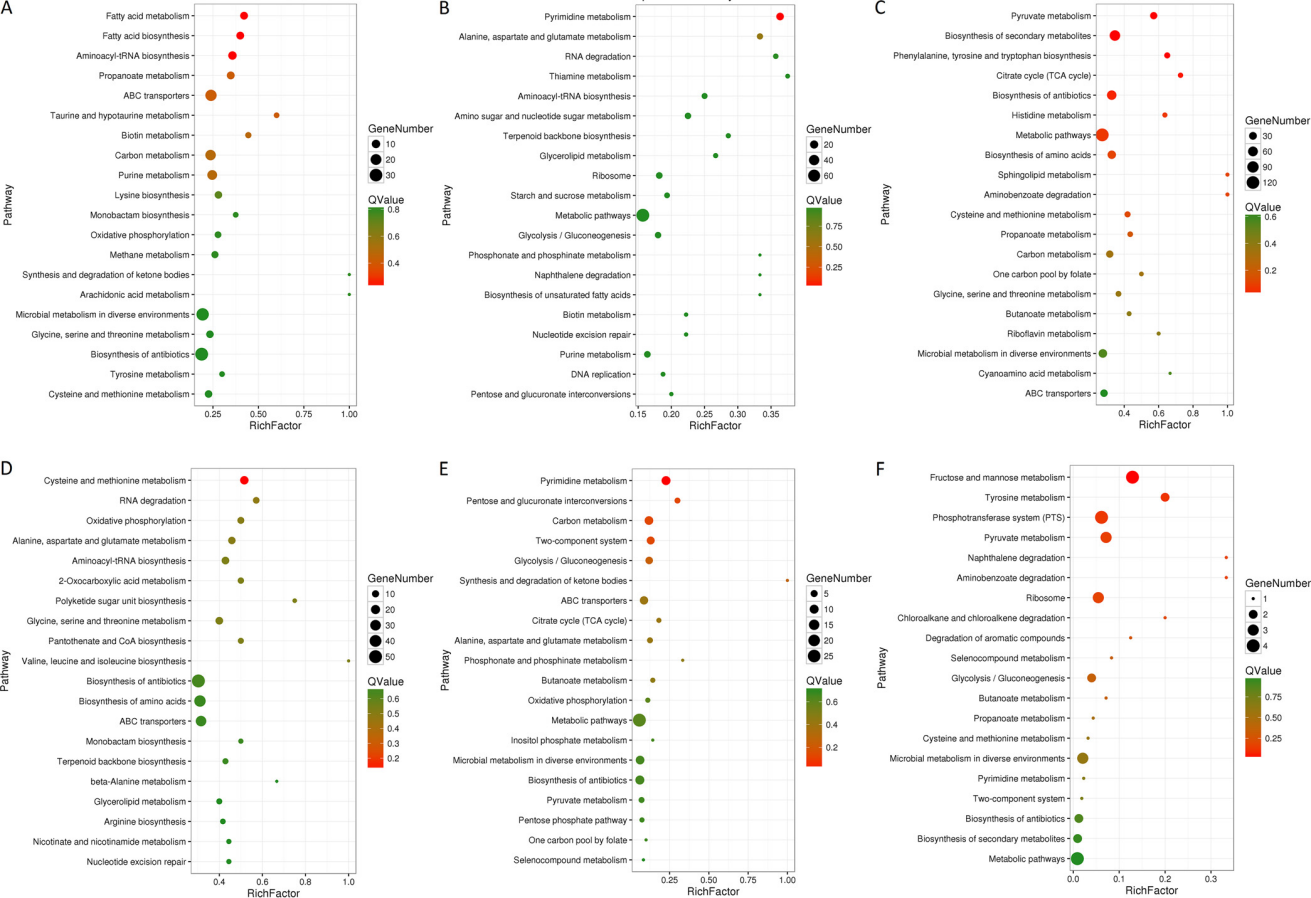
Top 20 enriched KEGG pathways in L. plantarum comparison groups. (A) Specific in Lh_12h vs LhSc_12h, (B) Specific in Lh_24h vs LhSc_24h, (C) Shared in Lh_12h vs LhSc_12h VS Lh_24h vs LhSc_24h, (D) Specific in Lh_12h vs Lh_24h, (E) Specific in LhSc_12h vs LhSc_24h, (F) Shared in Lh_12h vs Lh_24 h VS LhSc_12h vs LhSc_24h. RNA-seq was conducted in biological triplicates.

Concerning the L. plantarum samples, 4 comparative groups including Lh_12h vs Lh_24h, Lh_12h vs LhSc_12h, Lh_24h vs LhSc_24h, and LhSc_12h vs LhSc_24h were analyzed with DEGs identified (Fig. 7A to D). The DEGs in each group were further compared to identify specific and shared DEGs (Fig. 7E and F). Specific DEGs in one group was designated as the genes were not DEGs in any other groups. In Lh_12h vs LhSc_12h VS Lh_24h vs LhSc_24h group, 463 and 376 specific DEGs representing the key genes for interaction in 0 to 12 h and 12 to 24 h were identified in Lh_12h vs LhSc_12h and Lh_24h vs LhSc_24h groups, respectively, and 558 shared DEGs representing key genes for interaction in 0 to 24 h were identified (Fig. 7E). In Lh_12h vs Lh_24h VS LhSc_12h vs LhSc_24h group, 718 and 65 specific DEGs were identified in Lh_12h vs Lh_24h and LhSc_12h vs LhSc_24h groups, respectively, and 31 shared DEGs were identified (Fig. 7F). The specific genes in comparative group LhSc_12h vs LhSc_24h, representing the key genes required for L. plantarum to inhibit S. cerevisiae growth, were emphasized on in further analysis. KEGG pathway enrichment were performed on upregulated and downregulated DEGs respectively (Fig. 8).

### Key genes and pathways in L. plantarum.

Based on the changes in phenotypes including the inhibition on S. cerevisiae growth, the DEGs and respective enriched KEGG pathways in the 4 comparative groups of L. plantarum were analyzed to acquire the genes and pathways playing major roles in the interaction of L. plantarum with S. cerevisiae. Firstly, *lamB* (5.65 fold), *lamD* (6.12 fold), *lamC* (7.96 fold), *lamA* (11.96 fold) genes in *lamBDCA* quorum sensing (QS) system were specifically upregulated in Lh_24h vs LhSc_24h group, indicating the activation of *lam* QS system in coculture at 24 h. However, the *lamBDCA* QS system was inactivated in coculture at 12 h as these genes were not DEGs in Lh_12h vs LhSc_12h. Thus, the L. plantarum might reach the cell density threshold after 12 h and activate *lamBDCA* QS system under the external stimulation of S. cerevisiae cells. Secondly, besides the genes involved in the QS system, signaling factors were upregulated in groups Lh_12h vs LhSc_12h (*citF*, *citE*, *mae*, *citD*, *ifcA*, *mleS*, *isaA *× 2, *iap*) and Lh_24h vs LhSc_24h (*zmp1*, *pstF*, *pstE*, *dnaA*, *hpk3*, *ifcA*, *mleS*, *isaA *× 2, *iap*). In addition, *pstE* and *pstF* were specific upregulated genes in group LhSc_12h vs LhSc_24h, indicating they might be key signaling factors inducing the change in S. cerevisiae growth. Thirdly, partial metabolism and transport pathways were changed during coculture (Fig. 8), including the upregulation of pyruvate, propionic acid, carbohydrate metabolism pathways and downregulation of amino acid (cysteine, methionine, histidine, glycine, serine and threonine), purine, fatty acid metabolism pathways in 12 h, and upregulation of pyruvate metabolism pathway and downregulation of amino acid (phenylalanine, tyrosine, tryptophan, alanine, aspartic acid and glutamic acid) metabolism pathway in 24 h.

### General features of the transcriptome profile of S. cerevisiae.

**FIG 9:**
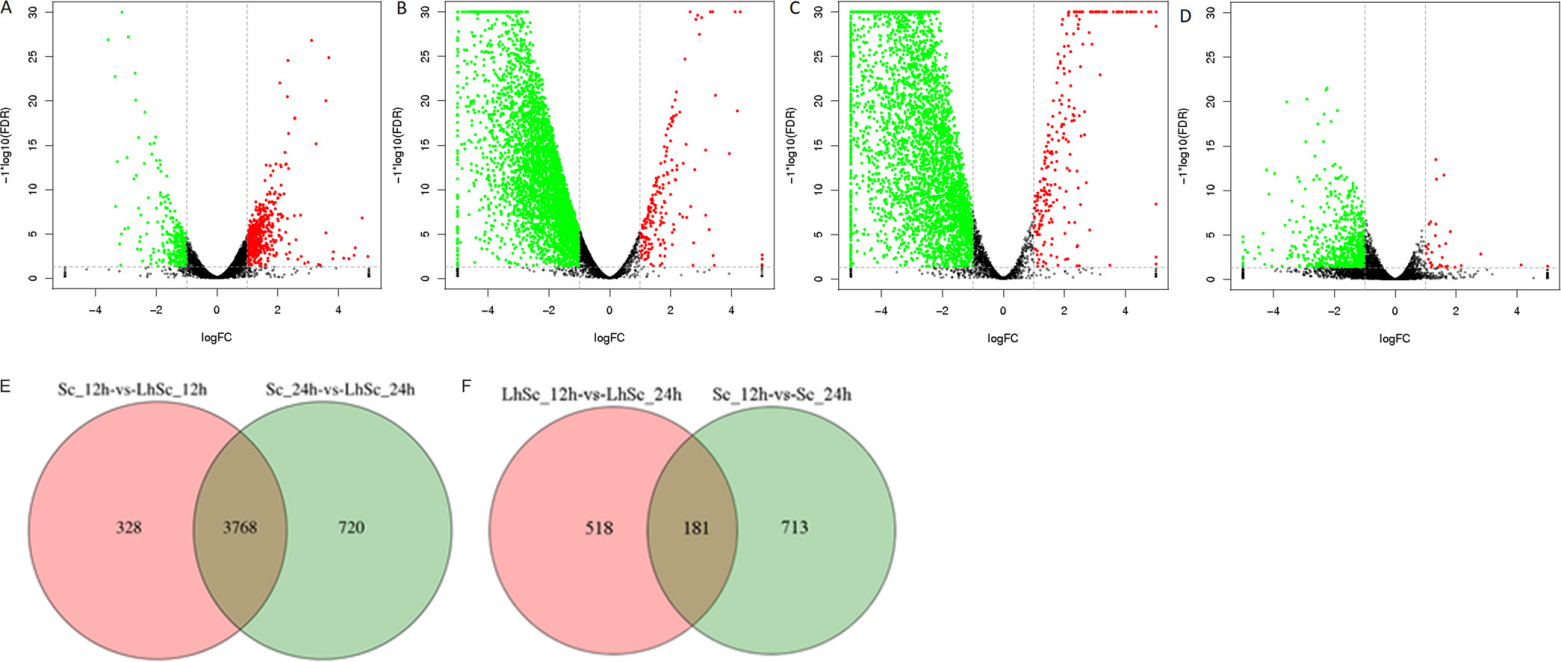
Volcano plots (A) to (D) and Venn map (E) to (F) of S. cerevisiae comparison groups. (A) Sc_12h vs Sc_24h, (B) Sc_12h vs LhSc_12h, (C) Sc_24h vs LhSc_24h, (D) LhSc_12h vs LhSc_24h. RNA-seq was conducted in biological triplicates.

**FIG 10:**
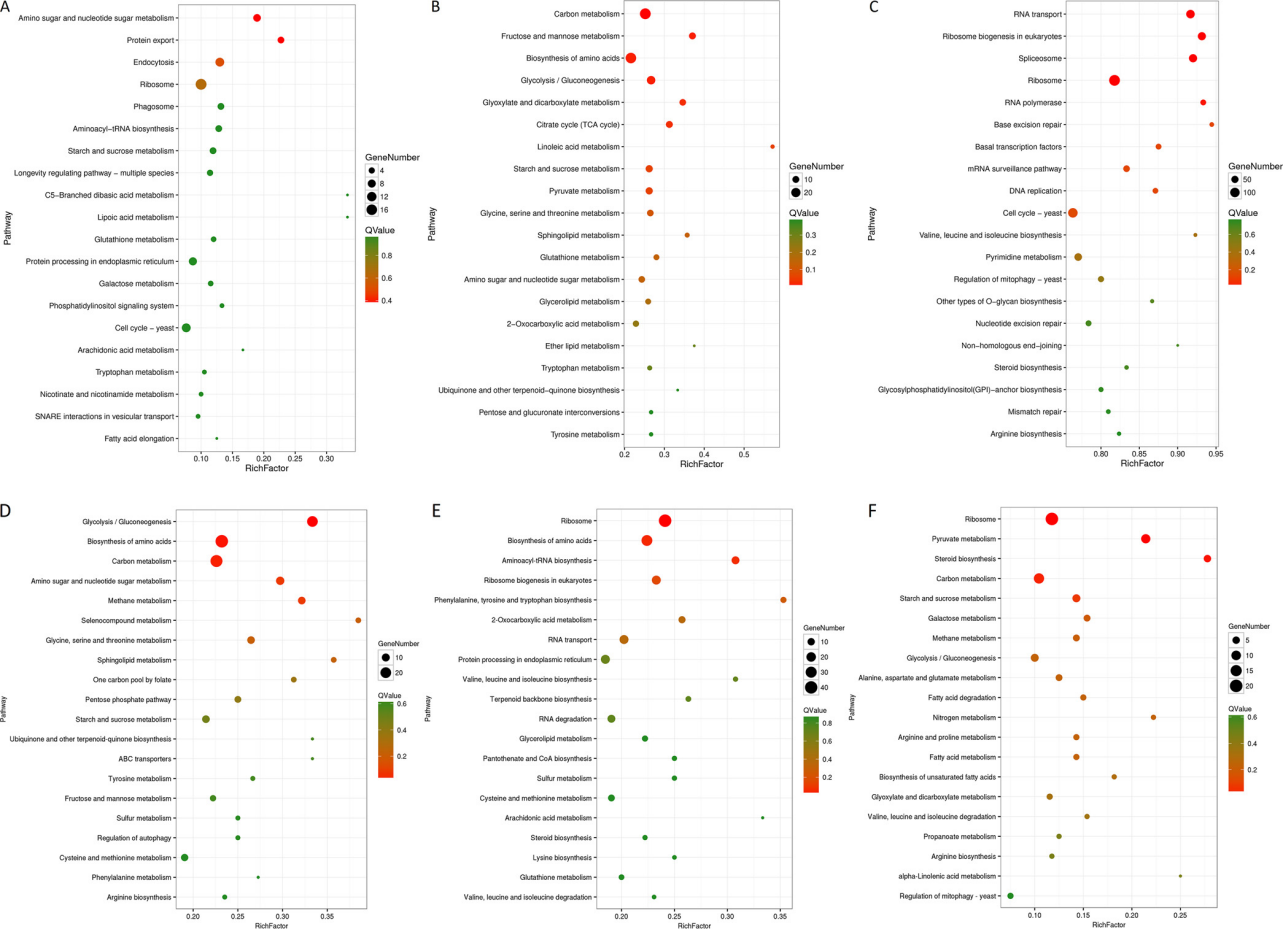
Top 20 enriched KEGG pathways in S. cerevisiae comparison groups. (A) Specific in Sc_12h vs LhSc_12h, (B) Specific in Sc_24h vs LhSc_24h, (C) Shared in Sc_12h vs LhSc_12h VS Sc_24h vs LhSc_24h, (D) Specific in Sc_12h vs Sc_24h, (E) Specific in LhSc_12h vs LhSc_24h, (F) Shared in Sc_12h vs Sc_24 h VS LhSc_12h vs LhSc_24h. RNA-seq was conducted in biological triplicates.

Concerning the S. cerevisiae samples, 4 comparative groups including Sc_12h vs Sc_24h, Sc_12h vs LhSc_12h, Sc_24h vs LhSc_24h, and LhSc_12h vs LhSc_24h were analyzed with DEGs identified (Fig. 9A to D). The DEGs in each group were further compared to identify specific and shared DEGs (Fig. 9E and F). In Sc_12h vs LhSc_12h VS Sc_24h vs LhSc_24h group, 328 and 720 specific DEGs representing the key genes for interaction in 0 to 12 h and 12 to 24 h were identified in Sc_12h vs LhSc_12h and Sc_24h vs LhSc_24h groups, respectively, and 3768 shared DEGs representing key genes for interaction in 0 to 24 h were identified (Fig. 9E). In Sc_12h vs Sc_24h VS LhSc_12h vs LhSc_24h group, 713 and 518 specific DEGs were identified in Sc_12h vs Sc_24h and LhSc_12h vs LhSc_24h groups, respectively, and 181 shared DEGs were identified (Fig. 9F). The specific genes in comparative group LhSc_12h vs LhSc_24h, representing the key genes related to the inhibition of S. cerevisiae growth, were emphasized on in further analysis. KEGG pathway enrichment were performed on upregulated and downregulated DEGs respectively (Fig. 10).

### Key genes and pathways in S. cerevisiae.

Given the changes in phenotypes including reduced growth and lowered surface roughness, the DEGs and respective enriched KEGG pathways in the 4 comparative groups of S. cerevisiae were analyzed to acquire the genes and pathways playing major roles in the interaction of S. cerevisiae with L. plantarum. Firstly, a few stress-response related genes were upregulated. In group Sc_12h vs LhSc_12h, 19 stress response genes including the response to oxidative stress, external biological stimulation, abiotic stimulation, osmotic stress, acidic pH, ethanol, and nutritional environment were upregulated. Among those, 15 (*PAU19*/*21*/*22*/*3*, *SRX1*, *GPX2*, *PDR15*, *PHM8*, *HSP104*, *RSB1*, *CIN5*, *DAK2*, *TRR1*, *HCH1*, *TPS2*) were specific DEGs in group Sc_12h vs LhSc_12h. In group Sc_24h vs LhSc_24h, 16 stress response genes including the response to oxidative stress, external biological stimulation, abiotic stimulation, osmotic stress, acidic pH, ethanol, nutritional environment, and DNA damage were upregulated. Among those, 11 (*BUD25*, *TEM1*, *OXR1*, *HTA2*, *TRX1*, *MXR2*, *HSC82*, *BCY1*, *HYR1*, *SCO1*, *THI4*) were specific DEGs in group Sc_24h vs LhSc_24h. Secondly, basic physiological activity and growth related pathways were downregulated in coculture, including protein export pathway in Sc_12h vs LhSc_12h, RNA transport, ribosome biogenesis, and ribosome pathways in Sc_24h vs LhSc_24h, and RNA transport, ribosome biogenesis, spliceosome, ribosomes, RNA polymerases, base excision repair, basal transcription factors, mRNA surveillance, DNA replication, post-downregulation of ribosomes, RNA polymerase, cell cycle, meiosis, and mitogen-activated protein kinase (MAPK) signaling pathways were shared in Sc_12h vs LhSc_12h and Sc_24h vs LhSc_24h. Thirdly, partial metabolism and transport pathways were influenced in coculture. Downregulation of amino sugar and nucleotide sugar, valine, leucine and isoleucine metabolic pathways in 12 h and glycolysis/glycogenous, 2-oxocarboxylic acid, amino acids (arginine, glycine, serine and threonine), carbohydrates (fructose, mannose, starch, sucrose), and pyruvate metabolic pathways in 24 h were identified (Fig. 10).

## DISCUSSION

The interaction between L. plantarum and S. cerevisiae had been reported to mainly rely on the signaling through extracellular metabolites (10, 16–18). In this study, L. plantarum and S. cerevisiae cells were cocultured in different initial concentrations to simulate their co-existence in the food system. L. plantarum inhibited S. cerevisiae growth since 12 h with its own growth pattern unaffected, implying the cell-cell interaction happened after 12 h coculture. Cell-cell direct contact was observed with L. plantarum cells surrounding S. cerevisiae cells. Co-aggregation had been previously identified between L. plantarum and S. cerevisiae (23). Such physical aggregation was normally short-term gathering to exchange metabolites or signaling factors (24). However, the aggregation between L. plantarum and S. cerevisiae in this study lasted for more than 12 h, indicating further genetic effects based on initial physical aggregation. In addition, the study on the effect of cell-free culture supernatant of L. plantarum on S. cerevisiae showed 24 h mature culture supernatant was capable of inhibit S. cerevisiae growth, independent of pH value. But the 12 h culture supernatant was insufficient to induce the reduced S. cerevisiae growth. Thus, the metabolites accumulated by L. plantarum during 12 h to 24 h were critical to the inhibition on S. cerevisiae growth. Moreover, with the same conditions including initial concentration and culturing time, L. plantarum and S. cerevisiae coculture supernatant showed stronger inhibition on S. cerevisiae growth than L. plantarum monococulture supernatant, suggesting the metabolism of both strains was changed during coculture. Significantly differential expressions of genes involved in metabolism and transport pathways were correlated with such changes.

Regarding cell-cell microbial interaction, besides physical interaction, activation of QS system is a major communication strategy among cells. QS is a process of cell-to-cell chemical communication that relies on the production, detection, and response to extracellular signaling molecules called autoinducers. Microbes typically integrate information encoded in QS autoinducers into the control of gene expression, which enables intra-species, intra-genera and inter-species communication (25). In L. plantarum, 2 QS systems including a 2-component regulatory system (TCS) and *luxS*/AI-2 signaling system has been identified. Two homologous *lamBDCA* and *lamKR* TCS cooperatively control adherence, cell morphology, and cell viability properties in L. plantarum (26, 27). In our study, *lamB*, *lamD*, *lamC*, and *lamA* genes in the *lamBDCA* QS system were specifically upregulated in coculture at 24 h. The activation of the *lamBDCA* QS system might play a critical role in the microbial interaction between L. plantarum and S. cerevisiae. The *lamBDCA* QS system encodes the 2-component histidine protein kinase lamC and response regulator lamA, an autoinducing peptide (AIP) derived from precursor peptide lamD, and additionally lamB, a protein that is involved in processing and posttranslational modification of lamD (27). Upon the processing and modification of lamB, lamD produces AIP to stimulate the phosphorylation of lamA by lamC. Phosphorylated lamA regulates the downstream gene expression, including surface polysaccharides, cell membrane proteins, sugar utilization proteins, and pyrimidine biosynthesis related genes (27). The activation of the *lamBDCA* QS system might be correlated with the changes in the metabolism of L. plantarum and stimulation on S. cerevisiae. In addition, *lamA* mutant had significantly decreased adherence, showing its significant role in cell adherence ability of L. plantarum (27). The accumulation of L. plantarum cells around S. cerevisiae cells in this study might attribute to the significant upregulation of the *lamA* gene.

Upon the stimulation of L. plantarum cells, potentially through the activation of *lamBDCA* QS system, S. cerevisiae upregulated stress response genes to overcome the oxidative stress, nutrient competition, biotic stimulation by cell-cell direct contact, and abiotic chemical stimulation by metabolites. Meanwhile, basic physiological activity (DNA replication, RNA transport, base excision repair, basal transcriptional factor, mRNA surveillance, protein processing, and protein export) and growth (ribosome biogenesis, cell cycle, meiosis, and MAPK signaling pathways) related pathways in S. cerevisiae were downregulated, which potentially result in partial cell death and lower growth rate. The protein export pathway regulates the active transport of proteins from the cytoplasm to the exterior of the cell (28). The downregulation of protein export pathway revealed possibly decreased protein export capability of S. cerevisiae in coculture. Furthermore, ribosomes are the cellular factories responsible for making proteins. In eukaryotes, including S. cerevisiae, ribosome biogenesis involves the production and correct assembly of 4 rRNAs and about 80 ribosomal proteins. In the absence of adequate gene expression and protein synthesis, ribosome biogenesis is stalled and cell growth is terminated (29). Therefore, downregulation of the ribosome biogenesis pathway in S. cerevisiae in coculture was corelated with its decreased growth rate. Strikingly, all genes involved in 4 MAPK signaling pathways (pheromone response pathway, filamentous growth pathway, high osmolarity/glycerol pathway, and cell wall integrity pathway) in S. cerevisiae showed significant downregulation in coculture.

The pheromone MAPK module (Ste11- Ste7 – Fus3), activated by cell-type-specific mating pheromones, induces remodeling of the cytoskeleton and cell wall, and eventually causes cell fusion with the mating partner (30). In this study, downregulation of the pheromone MAPK module in coculture indicated a lower level of mating pheromones in the extracellular environment, meaning less cell mating might happen, potentially leading to a reduced cell number. Under limited nutritional conditions, yeast cells undergo morphological changes and become more elongated and proliferate in a unipolar pattern called filamentous growth (31). The filamentous growth MAPK module (Ste11 - Ste7 - Kss1) is activated when carbon or nitrogen is limiting and controls cell adhesion, cell elongation, and the reorganization of cell polarity (30). In this study, no filamentous growth was observed in both mono- and coculture, in combination with the downregulation of filamentous growth MAPK module in S. cerevisiae upon coculture with L. plantarum for 24 h, suggesting the sufficient nutrition within 24 h. Besides the downregulation of the filamentous growth MAPK module, *msb2* gene showed 2.8-fold and 5.2-fold decrease in Sc_12h vs LhSc_12h and Sc_24h vs LhSc_24h at 24 h, respectively. Processing and release of the inhibitory extracellular glycodomain of Msb2p lead to activation of the filamentous growth MAPK pathway (32). *Msb2* is also an osmosensor activating high osmolarity/glycerol MAPK pathway in response to osmolarity change (33). Thus, the existence of L. plantarum cells might somehow downregulate the *msb2* gene through the alteration of environmental osmolarity and cause the inactivation of downstream filamentous growth and high osmolarity/glycerol MAPK pathways. Higher extracellular osmolarity level has been previously suggested to be sufficiently deleterious to threaten cell viability in the absence of a mechanism to restore osmotic balance (30). To increase the intracellular osmolarity to combat external hypertonic stress, yeast cells increase their synthesis of glycerol through the high osmolarity/glycerol MAPK pathway (31). In coculture with L. plantarum, S. cerevisiae might lose partial high osmolarity response capability and further cell viability due to the downregulation of the high osmolarity/glycerol MAPK pathway. It has been reported that the genes under control of the cell wall integrity MAPK pathway are involved in the synthesis and modification of the major components of the yeast cell wall (glucan, mannan, and chitin) (31). *Wsc1*, *Wsc2*, *Wsc3*, *Mid2*, and *Mtl1*, which have been identified as important for activation of the cell wall integrity pathway (34, 35), were significantly downregulated in the S. cerevisiae-L. plantarum coculture system. Hence, the S. cerevisiae cell wall integrity might be maintained in a lesser extent, as cell lysis was observed in coculture at 24 h. In addition, a few growth-essential genes, including *skg6*, *kex2*/*gas1*, and *ari1,* were downregulated during coculture. Skg6, an encoding transmembrane protein showing polarized intracellular localization, could suppress the synthetic lethality caused by a kex2/gas1 gene mutation (36). *Ari1* gene mutation has been identified to lower the growth rate of S. cerevisiae (37) The absent expression of *ari1* and *skg6*, together with the downregulation of *kex2*/*gas1*, might facilitate the depressed growth and proliferation rate of S. cerevisiae cells. Aside from activated stress response and weakened basal physiological activity and growth, partial metabolism and transport pathways were downregulated, including carbohydrates, amino acids, and pyruvate metabolism and transport in coculture. This could possibly prove the changes in metabolite accumulation in coculture supernatant.

### Conclusion.

**FIG 11:**
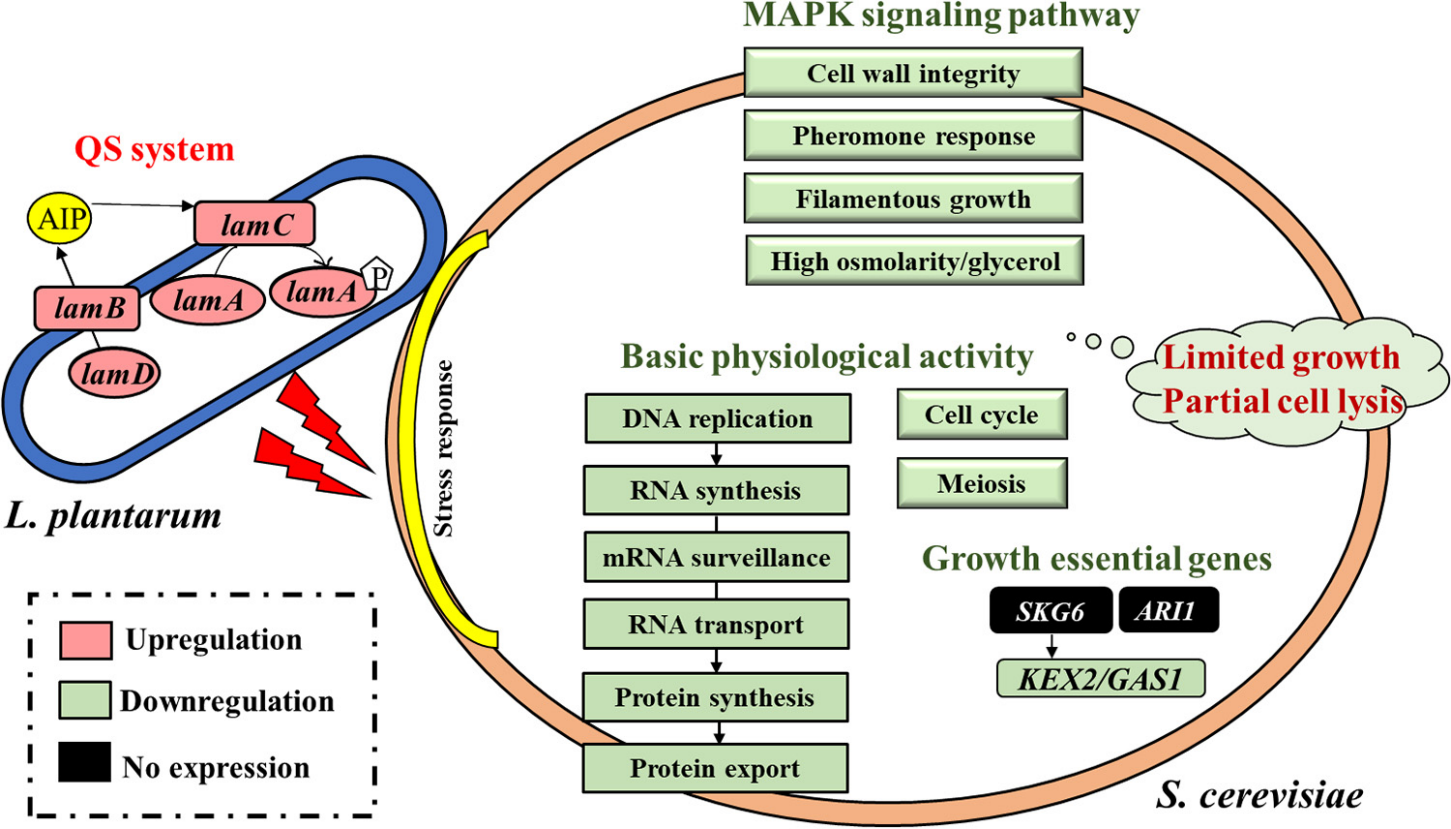
Hypothesis on the mechanism of microbial interaction between L. plantarum and S. cerevisiae.

In this study, microbial interaction between L. plantarum and S. cerevisiae was investigated in cell-cell and supernatant-cell model, with further transcriptome level mechanism elucidated. L. plantarum cells inhibited S. cerevisiae growth (inhibition rate ~80%) with its own growth pattern unaffected. Cell-cell aggregation happened during coculture with surface roughness changed and partial S. cerevisiae cell lysis. Mature (24 h) L. plantarum cell-free culture supernatant showed inhibition (35%-75%) on S. cerevisiae growth independent of pH level, while supernatant from L. plantarum-S. cerevisiae coculture showed relatively stronger inhibition. Upon transcriptomics analysis, hypothesis on the mechanism of microbial interaction between L. plantarum and S. cerevisiae was demonstrated (Fig. 11). When L. plantarum cell density reached a threshold at 24 h, all genes in the *lamBDCA* QS system were upregulated to potentially increase adhesion capability, leading to the aggregation to S. cerevisiae cells. Oxidative stress, biotic stimulation from cell-cell direct contact, and abiotic stimulation from metabolites might be posted on S. cerevisiae cells, potentially inducing the upregulation of stress response genes. However, on one hand, the downregulation of whole basic physiological activity from DNA to RNA to protein, cell cycle, meiosis, and pheromone MAPK pathway, as well as growth maintenance essential genes *ari1*, *skg6*, and *kex2*/*gas1* might induce the decreased growth and proliferation rate of S. cerevisiae cells. On the other hand, the depression of MAPK pathways including the filamentous growth pathway, high osmolarity/glycerol pathway, and cell wall integrity pathway might result in partial cell death. Hence, S. cerevisiae showed inhibited growth in coculture. In addition, metabolism pathways were altered in both strains. The results from this study demonstrated the antagonistic effect between L. plantarum and S. cerevisiae. Nevertheless, alteration in global transcriptomics profile for other *Lactobacillus* strains with none of inhibition on S. cerevisiae growth, requires further investigation.

## MATERIALS AND METHODS

### Microbial strains and growth conditions.

S. cerevisiae strain BM-SC17426 and L. plantarum strain BM-LP14723 were maintained as glycerol stock stored at −80°C. A small amount of S. cerevisiae stock was spread onto yeast peptone dextrose (YPD) agar and incubated at 30°C for 48 h to obtain single colonies. A single colony of S. cerevisiae was transferred to 2 mL of YPD broth and incubated at 30°C with shaking at 200 rpm overnight prior to further experiments. A small amount of L. plantarum stock was spread onto De Man Rogosa Sharpe (MRS) agar (Oxoid) and incubated at 37°C for 48 h to obtain single colonies. A single colony of L. plantarum was transferred to 2 mL of MRS broth and incubated at 37°C with shaking at 200 rpm overnight prior to further experiments.

### Cell-cell coculture model.

To investigate the potential interaction between S. cerevisiae and L. plantarum cells with different initial concentration, a cell-cell coculture model was set up. Overnight culture cells of S. cerevisiae and L. plantarum strains were washed twice with phosphate-buffered saline (PBS) and diluted to 10^7^ or 10^5^ CFU/mL by YPD and MRS broth, respectively. One mL each of S. cerevisiae and L. plantarum cultures were mixed with a concentration ratio of 1:100, 1:1, and 100:1, respectively, as coculture group. One mL each of L. plantarum culture and YPD broth were mixed as L. plantarum monococulture group. One mL each of S. cerevisiae culture and MRS broth were mixed as S. cerevisiae monococulture group. One mL each of distilled H_2_O and S. cerevisiae culture were mixed as S. cerevisiae blank control group. One mL each of distilled H_2_O and L. plantarum culture were mixed as L. plantarum blank control group. Three experimental groups were incubated at 37°C with shaking at 200 rpm. Cell numbers were determined by CFU counting on selective agar plates every 4 h. Cells in coculture group and L. plantarum monococulture group were numerated on MRS agar supplemented with 10 μg/mL amphotericin B (Shanghai yuanye Bio-Technology Co., Ltd). Cells in coculture group and S. cerevisiae monococulture group were numerated on YPD agar supplemented with 200 μg/mL chloramphenicol (Shanghai yuanye Bio-Technology Co., Ltd). The experiments were performed in 3 biological replicates.

### Supernatant-cell coculture model.

Considering the difference of metabolites in monococulture and coculture groups, supernatant from L. plantarum monococulture and S. cerevisiae and L. plantarum coculture were collected to determine the effect on S. cerevisiae growth. Upon monococulture supernatant collection, overnight culture cells of L. plantarum were washed twice with PBS and diluted to 10^7^ or 10^5^ CFU/mL by MRS broth, followed by incubating at 37°C with shaking at 200 rpm for 6 h, 12 h, 24 h, and 48 h, respectively. As to coculture supernatant collection, S. cerevisiae and L. plantarum coculture group was set up as mentioned above and incubated for 6 h, 12 h, 24 h, and 48 h, respectively. Collected mono- and coculture were centrifuged at 5,000 rpm for 1 min and supernatants were pipetted out and filtered through 0.22 μm microfilter (Millipore). The pH values of mono- and coculture supernatants were determined using a PHS-3C pH indicator (Shanghai puchun Measure Instrument Co., Ltd.).

One mL each of L. plantarum monococulture supernatant and S. cerevisiae culture were mixed as mono-supernatant-cell coculture group. One mL each of MRS broth and S. cerevisiae culture were mixed as mono-supernatant control group. One mL each of pH adjusted MRS broth and S. cerevisiae culture were mixed as pH adjusted mono-supernatant control group. One mL each of coculture supernatant and S. cerevisiae culture were mixed as co-supernatant-cell coculture group. One mL each of MRS-YPD broth mixture (1:1) and S. cerevisiae culture were mixed as co-supernatant control group. One mL each of pH adjusted MRS-YPD broth mixture (1:1) and S. cerevisiae culture were mixed as pH adjusted co-supernatant control group. One mL each of distilled H_2_O and S. cerevisiae culture were mixed as blank control group. One mL each of pH adjusted distilled H_2_O and S. cerevisiae culture were mixed as pH adjusted blank control group. All experimental groups were incubated at 37°C with shaking at 200 rpm. OD_600_ value was determined every 2 h using InfiniteM200 Pro multimode plate reader (Tecan) and CFU counting on selective YPD agar was performed at specific time points. The experiments were performed in 3 biological replicates.

### Morphology observation by optical and atomic force microscopes.

In the cell-cell coculture model, 12 h and 24 h coculture and monococulture samples were collected and observed under Axioskop 40 pol optical microscope (Zeiss). The samples were collected from 3 biological replicates and at least 20 images were taken for each sample. For the determination of cell surface roughness, the samples were firstly scraped with a sterile blade to expose the substrate. Afterwards, the probe was used to approach and make contact with the cells and the substrate using AFM XE-100 (Park Systems). AFM performed in non-contact mode was used to characterize the morphology of individual cells in air (38). Silicon cantilever (PPP-NCHR, Nanosensors) with spring constant of 42 N/m and resonance frequency of 330 kHz was used. The scan rate was 0.5 Hz and the image resolution was 256 pixels × 256 pixels. Data were recorded for at least 10 fields of view per sample and results represent typical observation in each field. Surface roughness was determined using XEI software.

### RNA isolation, library construction, and sequencing.

In the cell-cell coculture model, 12 h and 24 h coculture and monococulture samples were collected from 3 biological replicates and fast frozen using liquid nitrogen. Total RNA was extracted using TRIzol reagent (Sigma-Aldrich) based on manufacturer’s instruction. Eukaryotic mRNA was enriched by Oligo(dT) beads, while prokaryotic mRNA was enriched by removing rRNA by Ribo Zero Magnetic Kit (Epicentre). The mRNA was fragmented using fragmentation buffer and was then reverse transcribed to cDNA with random primers. Second strand cDNA were synthesized by DNA polymerase I, RNase H, dNTP and buffer. cDNA fragments were purified with QiaQuick PCR extraction kit, end repaired, poly(A) added, and ligated to Illumina sequencing adapters. The ligation products were size selected by agarose gel electrophoresis, PCR amplified, and sequenced using Illumina HiSeq 2500 by Gene Denovo Biotechnology Co. (Guangzhou).

### Differentially expressed genes identification and annotation.

Raw reads obtained from the sequencing platform included adapters or low quality affecting following assembly and analysis. Thus, reads containing adapters, more than 10% of unknown nucleotides (N), and more than 50% of low quality (Q value ≤ 20) bases were filtered to get high quality clean reads. Reads aligned to rRNA were analyzed in a database by a short reads alignment tool Bowtie 2 (39) were also removed. Clean reads were quality examined by FastQC v.0.10.1 (http://www.bioinformatics.babraham.ac.uk/projects/fastqc/) and aligned to reference genome using TopHat2 (version 2.0.3.12) (40). No more than 2 bases mismatch and read gap were allowed in the alignment. The genomes of S. cerevisiae strain S288C and L. plantarum strain WCFS1 were used as reference genomes. The transcriptomes of L. plantarum monococulture samples were 90.96% to 94.53% mapped to the reference genome, while the transcriptomes of S. cerevisiae monococulture samples were 86.38% to 89.19% mapped to the reference genome.

Gene abundance was quantified by software RSEM (41). Gene expression level was normalized by the Fragments Per Kilobase of transcript per Million mapped reads (FPKM) method. To identify differentially expressed genes (DEGs) between samples, the edge R package (http://www.r-project.org/) was used. Genes with fold change ≥ 2 and false discovery rate (FDR) <0.05 in a comparative group were considered as significant DEGs. DEGs were then subjected to enrichment analysis of KEGG pathways (42). The significantly enriched KEGG pathways were identified by *P* value < 0.05 in Fisher Exact Test and *P* value < 0.01 in Hypergeometric Distribution, respectively, and adjusted by FDR (43).

### Statistical analysis.

The experimental data were shown as the mean ± standard deviation (SD) from at least 3 replicates. Statistical differences among the results obtained by the treatments and control were examined by Student's *t* test and one-way analysis of variance (ANOVA), followed by a Tukey multiple intergroup comparison, where appropriate. A value of *P* < 0.05 was considered statistically significant.

### Data availability.

The RNA-seq clean data obtained from the mono- and coculture samples of L. plantarum and S. cerevisiae in this study have been submitted to the NCBI database under BioProject ID PRJNA832027.
